# Supplementary material for: Proactive and reactive engagement of artificial intelligence methods for education: a review
Source: Front Artif Intell. 2023 May 5;6:1151391. doi: 10.3389/frai.2023.1151391 (PMC10196470; doi:10.3389/frai.2023.1151391)
Supplement: Supplementary file 1 [file Data_Sheet_1.PDF]

Supplementary Material

1 SUPPLEMENTARY TABLES AND FIGURES

Table S1: **List of technical articles reviewed in the paper.**

Category	Subcategories	Sl. No.	Paper Title
Student Admission Logistics		1	GRADE: Machine Learning Support for Graduate Admissions (Waters and Miikkulainen, 2014)
		2	A Quantitative Machine Learning Approach to Master Students Admission for Professional Institutions(Zhao et al., 2020)
		3	Applying machine learning to predict Davidson College's admissions yield (Jamison, 2017)
		4	Improve the Accuracy of Students Admission at Universities Using Machine Learning Techniques (Assiri et al., 2022)
		5	A machine learning approach for graduate admission prediction (AlGhamdi et al., 2020)
		6	Graduate admission chance prediction using deep neural network (Goni et al., 2020)
		7	Machine Learning Algorithms for Predicting the Graduation Admission (Mridha et al., 2022)
		8	Using machine learning to understand physics graduate school admissions (Young and Caballero, 2019)
		9	A Recommender System for Predicting Students' Admission to a Graduate Program using Machine Learning Algorithms (El Guabassi et al., 2021)
Content Design	Learning Content Design	10	Applying machine learning to improve curriculum design (Ball et al., 2019)
		11	Application of machine learning to curriculum design analysis (Rawatlal, 2017)
		12	Curriculum design using artificial intelligence (AI) back propagation method (Somasundaram et al., 2020)
		13	Integrating human and machine intelligence for enhanced curriculum design (Doroudi, 2019)
		14	Linguistic features to predict query difficulty (Mothe and Tanguy, 2005)
		15	Assessing scientific reasoning: A comprehensive evaluation of item features that affect item difficulty (Stiller et al., 2016)
		16	Introducing a framework to assess newly created questions with natural language processing (Benedetto et al., 2020a)
		17	Predicting item survival for multiple choice questions in a high-stakes medical exam (Yaneva et al., 2020)

		18	R2de: a nlp approach to estimating IRT parameters of newly generated questions (Benedetto et al., 2020b)
		19	Exercise difficulty prediction in online education system (Fang et al., 2019)
		20	Automated prediction of item difficulty in reading comprehension using long short-term memory (Lin et al., 2019)
		21	Predicting the difficulty and response time of multiple choice questions using transfer learning (Xue et al., 2020)
		22	Question difficulty prediction for reading problems in standard test (Huang et al., 2017)
		23	Question difficulty prediction for multiple choice problems in medical exams (Qiu et al., 2019)
		24	Stan: Adversarial network for cross-domain question difficulty prediction (Huang et al., 2021)
	Timetabling	25	Incorporating machine learning to evaluate solutions to the university course timetabling problem (Kenekayoro, 2019)
		26	Non-linear great deluge with reinforcement learning for university course timetabling (Obit et al., 2011)
		27	A reinforcement learning: great-deluge hyper-heuristic for examination timetabling (Özcan et al., 2012)
		28	Simulated annealing with improved reheating and learning for the post enrolment course timetabling problem (Goh et al., 2019)
Content Generation		29	Automatic gap-fill question generation from text books (Agarwal and Mannem, 2011)
		30	Automatic factual question generation from text (Heilman, 2011)
		31	Generating natural language questions to support learning on-line (Lindberg et al., 2013)
		32	Natural language question generation using syntax and keywords (Kalady et al., 2010)
		33	A system for generating multiple choice questions: With a novel approach for sentence selection (Majumder and Saha, 2015)
		34	Automatic generation of cloze question stems (Correia et al., 2012)
		35	Good question! statistical ranking for question generation (Heilman and Smith, 2010)
		36	Learning to ask: Neural question generation for reading comprehension (Du et al., 2017)
		37	Paragraph-level neural question generation with maxout pointer and gated self-attention networks (Zhao et al., 2018)
		38	Improving neural question generation using answer separation (Kim et al., 2019)
		39	Reinforcement learning based graph-to-sequence model for natural question generation (Chen et al., 2020)
		40	Building a semantic open learning space with adaptive question generation support (Jouault and Seta, 2013)

		41	Generating natural language question-answer pairs from a knowledge graph using a RNN based question generation model (Indurthi et al., 2017)
		42	Generating natural questions about an image (Mostafazadeh et al., 2016)
		43	Tedquiz: automatic quiz generation for ted talks video clips to assess listening comprehension (Huang et al., 2014)
		44	Automatic item generation via frame semantics: Natural language generation of math word problems (Deane and Sheehan, 2003)
		45	Personalized mathematical word problem generation (Polozov et al., 2015)
		46	A theme-rewriting approach for generating algebra word problems (Koncel-Kedziorski et al., 2016)
		47	Towards generating math word problems from equations and topics (Zhou and Huang, 2019)
		48	Mathematical word problem generation from commonsense knowledge graph and equations (Liu et al., 2021)
		49	Math word problem generation with mathematical consistency and problem context constraints (Wang et al., 2021)
		50	Automatic math word problem generation with topic-expression co-attention mechanism and reinforcement learning (Wu et al., 2022)
Tutoring aids	Interactive tutoring aids	51	Wayang outpost: Intelligent tutoring for high stakes achievement tests (Arroyo et al., 2004)
		52	Guru: A computer tutor that models expert human tutors (Olney et al., 2012)
		53	Sociable robot improves toddler vocabulary skills (Movellan et al., 2009)
		54	Bayesian active learning based robot tutor for children's word-reading skills (Gordon and Breazeal, 2015)
		55	Leveraging chatbots to improve self-guided learning through conversational quizzes (Pereira, 2016)
		56	AI Medical School Tutor: Modelling and Implementation (Afzal et al., 2020)
		57	Learning a Skill-Teaching Curriculum with Dynamic Bayes Nets (Green et al., 2011)
		58	Automated Geometry Theorem Proving for Human-Readable Proofs (Wang and Su, 2015)
		59	Hybrid Conversational AI for Intelligent Tutoring Systems (Pande et al., 2021)
		60	Intelligent Virtual Reality Tutoring System Supporting Open Educational Resource Access (Ahn et al., 2018)
		61	The Virtual Operative Assistant: An explainable artificial intelligence tool for simulation-based training in surgery and medicine (Mirchi et al., 2020)

		62	A Design Proposition for Interactive Virtual Tutors in an Informed Environment (Taoum et al., 2016)
		63	Branching Storylines in Virtual Reality Environments for Leadership Development (Gordon et al., 2004)
	Personalized tutoring aids	64	Adaptive Course Sequencing for Personalization of Learning Path Using Neural Network (Idris et al., 2009)
		65	Data mining for providing a personalized learning path in creativity: An application of decision trees (Lin et al., 2013)
		66	Accelerating human learning with deep reinforcement (Reddy et al., 2017)
		67	Deep reinforcement learning of marked temporal point processes (Upadhyay et al., 2018)
		68	A Deep Reinforcement Learning Framework for Instructional Sequencing (Pu et al., 2020)
		69	PAKES: A Reinforcement Learning-Based Personalized Adaptability Knowledge Extraction Strategy for Adaptive Learning Systems (Islam et al., 2021)
		70	Pedagogical discourse: connecting students to past discussions and peer mentors within an online discussion board (Kim and Shaw, 2009)
		71	A deep learning-based course recommender system for sustainable development in education (Li and Kim, 2021)
	Affect aware tutoring aids	72	A multimedia adaptive tutoring system for mathematics that addresses cognition, metacognition and affect (Arroyo et al., 2014)
		73	The effect of motivational learning companions on low achieving students and students with disabilities (Woolf et al., 2010)
		74	Bayesian networks and linear regression models of students' goals, moods, and emotions (Arroyo et al., 2010)
		75	Detecting and addressing frustration in a serious game for military training (DeFalco et al., 2018)
		76	Improving Sensor-Free Affect Detection Using Deep Learning (Botelho et al., 2017)
	Learning style aware tutoring aids	77	Identification of learning styles online by observing learners' browsing behaviour through a neural network (Lo and Shu, 2005)
		78	Learning styles' recognition in e-learning environments with feed-forward neural networks (Villaverde et al., 2006)
		79	Online Learning Styles Identification Model, Based on the Analysis of User Interactions Within an E-Learning Platforms, Using Neural Networks and Fuzzy Logic (Alfaro et al., 2018)
		80	Smart Education with artificial intelligence based determination of learning styles (Bajaj and Sharma, 2018)
		81	A learning social network with recognition of learning styles using neural networks (Zatarain-Cabada et al., 2010)

		82	Using artificial neural networks to identify learning styles (Bernard et al., 2015)
		83	Using learning styles and neural networks as an approach to elearning content and layout adaptation (Mota, 2008)
Performance assessment and monitoring	Student-focused	84	Learning factors analysis–a general method for cognitive model evaluation and improvement (Cen et al., 2006)
		85	Performance factors analysis–a new alternative to knowledge tracing (Pavlik Jr et al., 2009)
		86	Practice and forgetting effects on vocabulary memory: An activation-based model of the spacing effect (Pavlik Jr and Anderson, 2005)
		87	Individualized bayesian knowledge tracing model (Yudelson et al., 2013)
		88	Integrating latent-factor and knowledge-tracing models to predict individual differences in learning (Khajah et al., 2014)
		89	Recommender system for predicting student performance (Thai-Nghe et al., 2010)
		90	Collaborative filtering applied to educational data mining (Toscher and Jahrer, 2010)
		91	Deep knowledge tracing (Piech et al., 2015a)
		92	Dynamic key-value memory networks for knowledge tracing (Zhang et al., 2017)
		93	Knowledge tracing with sequential key-value memory networks (Abdelrahman and Wang, 2019)
		94	A self-attentive model for knowledge tracing (Pandey and Karypis, 2019)
		95	Context-aware attentive knowledge tracing (Ghosh et al., 2020)
		96	Exercise-enhanced sequential modeling for student performance prediction (Su et al., 2018)
		97	Ekt: Exercise-aware knowledge tracing for student performance prediction (Liu et al., 2019)
		98	Augmenting knowledge tracing by considering forgetting behavior (Nagatani et al., 2019)
		99	Learning process-consistent knowledge tracing (Shen et al., 2021)
		100	Graph-based knowledge tracing: modeling student proficiency using graph neural network (Nakagawa et al., 2019)
		101	Gikt: a graph-based interaction model for knowledge tracing (Yang et al., 2020)
		102	Structure-based knowledge tracing: an influence propagation view (Tong et al., 2020)
		103	Learning to represent student knowledge on programming exercises using deep learning (Wang et al., 2017)

		104	Flexible domain adaptation for automated essay scoring using correlated linear regression (Phandi et al., 2015)
		105	Constrained multi-task learning for automated essay scoring (Cummins et al., 2016)
		106	Automated essay scoring with ontology based on text mining and nltk tools (Contreras et al., 2018)
		107	A neural approach to automated essay scoring (Taghipour and Ng, 2016)
		108	Augmenting textual qualitative features in deep convolution recurrent neural network for automatic essay scoring (Dasgupta et al., 2018)
		109	Automated essay scoring with discourse-aware neural models (Nadeem et al., 2019)
		110	Robust neural automated essay scoring using item response theory (Uto and Okano, 2020)
		111	Modeling organization in student essays (Persing et al., 2010)
		112	Modeling prompt adherence in student essay (Persing and Ng, 2014)
		113	Modeling thesis clarity in student essays (Persing and Ng, 2013)
		114	Modeling argument strength in student essays (Persing and Ng, 2015)
		115	Give me more feedback ii: Annotating thesis strength and related attributes in student essays (Ke et al., 2019)
		116	Sednn: Shared and enhanced deep neural network model for cross-prompt automated essay scoring (Li et al., 2020)
		117	Multi-stage pre-training for automated chinese essay scoring Song et al. (2020)
		118	Automated essay evaluation: The criterion online writing service (Burstein et al., 2004)
		119	Using natural language processing to provide formative feedback on text evidence usage in student writing (Zhang et al., 2019)
		120	Semantic similarity-based grading of student programs (Wang et al., 2007)
		121	Software verification and graph similarity for automated evaluation of students' assignments (Vujošević-Janičić et al., 2013)
		122	Syntactic and functional variability of a million code submissions in a machine learning mooc (Huang et al., 2013)
		123	Domain-independent proximity measures in intelligent tutoring systems (Mokbel et al., 2013)
		124	A system to grade computer programming skills using machine learning (Srikant and Aggarwal, 2014)
		125	Learning program embeddings to propagate feedback on student code (Piech et al., 2015b)

		126	Question independent grading using machine learning: The case of computer program grading (Singh et al., 2016)
		127	Use of machine learning methods in the assessment of programming assignments (Tarcsay et al., 2022)
		128	Zero shot learning for code education: Rubric sampling with deep learning inference (Wu et al., 2019)
		129	High performance automatic mispronunciation detection method based on neural network and trap features (Li et al., 2009)
		130	Detecting mispronunciations of L2 learners and providing corrective feedback using knowledge-guided and data-driven decision trees (Li et al., 2016)
		131	End-to-end automatic pronunciation error detection based on improved hybrid ctc/attention architecture (Zhang et al., 2020)
		132	Automatic spontaneous speech grading: A novel feature derivation technique using the crowd (Shashidhar et al., 2015)
		133	Mathematical language processing: Automatic grading and feedback for open response mathematical questions (Lan et al., 2015)
		134	Improving automated scoring of student open responses in mathematics (Baral et al., 2021)
		135	Clustering latex solutions to machine learning assignments for rapid assessment (Tan et al., 2017)
		136	Automatic assessment of student answers for geometric theorem proving questions (Mendis et al., 2017)
		137	Automatic short math answer grading via in-context meta-learning (Zhang et al., 2022)
		138	Arabic plagiarism detection using word correlation in n-grams with k-overlapping approach (Alzahrani, 2015)
		139	Using k-means cluster based techniques in external plagiarism detection (Vani and Gupta, 2014)
		140	Using natural language processing techniques and fuzzy-semantic similarity for automatic external plagiarism detection (Gupta et al., 2014)
		141	Glad: Groningen lightweight authorship detection (Hürlimann et al., 2015)
		142	Detecting plagiarism in text documents through grammar-analysis of authors (Tschuggnall and Specht, 2013)
		143	Using word embedding for cross-language plagiarism detection (Ferrero et al., 2017)
		144	Experiments on the indonesian plagiarism detection using latent semantic analysis (Soleman and Purwarianti, 2014)
		145	Analyzing semantic concept patterns to detect academic plagiarism
		146	Dls @ cu: Sentence similarity from word alignment (Meuschke et al., 2017) (Sultan et al., 2014)

		147	Detailed analysis of extrinsic plagiarism detection system using machine learning approach (naive bayes and svm) (Alfikri and Purwarianti, 2014)
		148	Exb themis: Extensive feature extraction from word alignments for semantic textual similarity (Hänig et al., 2015)
		149	Comparing and combining content-and citation-based approaches for plagiarism detection (Pertile et al., 2016)
		150	A new online plagiarism detection system based on deep learning (El Mostafa Hambi, 2020)
		151	Reliable plagiarism detection system based on deep learning approaches (El-Rashidy et al., 2022)
		152	A source code linearization technique for detecting plagiarized programs (Ji et al., 2007)
		153	Using code metric histograms and genetic algorithms to perform author identification for software forensics (Lange and Mancoridis, 2007)
		154	Efficient clustering-based source code plagiarism detection using piy (Ohmann and Rahal, 2015)
		155	An intelligent decision support system for software plagiarism detection in academia (Ullah et al., 2021)
		156	A deep learning framework for the detection of source code plagiarism using siamese network and embedding models (Manahi, 2021)
		157	Machine learning for source-code plagiarism detection (Katta, 2018)
		158	A fuzzy-based approach to programming language independent source-code plagiarism detection (Acampora and Cosma, 2015)
		159	A machine learning based tool for source code plagiarism detection (Bandara and Wijayarathna, 2011)
	Teacher-focused	160	A sentiment analysis model to analyze students reviews of teacher performance using support vector machines (Esparza et al., 2017)
		161	Mining: Students comments about teacher performance assessment using machine learning algorithms (Gutiérrez et al., 2018)
		162	Mining opinions from instructor evaluation reviews: a deep learning approach (Onan, 2020)
		163	Towards teaching analytics: a contextual model for analysis of students' evaluation of teaching through text mining and machine learning classification (Okoye et al., 2022)
		164	Predicting the performance of instructors using machine learning algorithms (Vijayalakshmi et al., 2020)
		165	Machine learning-based app for self-evaluation of teacher-specific instructional style and tools (Duzhin and Gustafsson, 2018)

		166	Analyzing teaching performance of instructors using data mining techniques (Mardikyan and Badur, 2011)
		167	Using data mining to predict instructor performance (Ahmed et al., 2016)
		168	Prediction of instructor performance using machine and deep learning techniques (Abunasser et al., 2022)
Outcome prediction	Apriori performance prediction	169	Estimating student retention and degree-completion time: Decision trees and neural networks vis-'a-vis regression (Herzog, 2006)
		170	A Comparative Analysis of Techniques for Predicting Academic Performance (Nghe et al., 2007)
		171	A Comparative Analysis of Techniques for Predicting Student Performance (Bydžovská, 2016)
		172	Deep Learning with Data Transformation and Factor Analysis for Student Performance Prediction (Dien et al., 2020)
		173	Modeling learner engagement in MOOCs using probabilistic soft logic (Ramesh et al., 2013)
		174	Predicting MOOC performance with week 1 behavior (Jiang et al., 2014)
		175	Predicting student risks through longitudinal analysis (Tamhane et al., 2014)
		176	Progressive Prediction of Student Performance in College Programs (Xu et al., 2017)
		177	A Study of Educational Data Mining: Evidence from a Thai University (Trakunphutthirak et al., 2019)
		178	How Widely Can Prediction Models be Generalized? (Gitinabard et al., 2019)
		179	Predicting academic performance of students from VLE big data using deep learning models (Waheed et al., 2020)
		180	Transfer Learning from Deep Neural Networks for Predicting Student Performance (Tsiakmaki et al., 2020)
		181	Predicting Students' Performance With School and Family Tutoring Using Generative Adversarial Network-Based Deep Support Vector Machine (Chui et al., 2020)
	Apriori attrition prediction	182	Predicting students drop out: A case study (Dekker et al., 2009)
		183	Who, When, and Why: A machine learning approach to prioritizing students at risk of not graduating high school on time (Aguiar et al., 2015)
		184	Bringing student backgrounds online: MOOC user demographics, site usage, and online learning (DeBoer et al., 2013)
		185	Engagement vs Performance: Using Electronic Portfolios to Predict First Semester Engineering Student Persistence (Aguiar et al., 2014)

		186	Learning latent engagement patterns of students in online courses (Ramesh et al., 2014)
		187	Predicting MOOC dropout over weeks using machine learning methods (Kloft et al., 2014)
		188	Capturing ”attrition intensifying” structural traits from didactic interaction sequences of MOOC learners (Sinha et al., 2014)
		189	Turn on, tune in, drop out: Anticipating student dropouts in massive open online courses (Yang et al., 2013)
		190	Sentiment Analysis in MOOC Discussion Forums: What does it tell us? (Wen et al., 2014)
		191	Identifying at-risk students in massive open online courses (He et al., 2015)
		192	A Machine Learning Framework to Identify Students at Risk of Adverse Academic Outcomes (Lakkaraju et al., 2015)
		193	A time series interaction analysis method for building predictive models of learners using log data (Brooks et al., 2015)
		194	Predicting student dropout in a MOOC: An evaluation of a deep neural network model (Imran et al., 2019)
		195	Predicting student dropout in subscription-based online learning environments: The beneficial impact of the logit leaf model (Coussement et al., 2020)

REFERENCES

Abdelrahman, G. and Wang, Q. (2019). Knowledge tracing with sequential key-value memory networks. In *Proceedings of the 42nd International ACM SIGIR Conference on Research and Development in Information Retrieval*. 175–184

Abunasser, B. S., AL-Hiealy, M. R. J., Barhoom, A. M., Almasri, A. R., and Abu-Naser, S. S. (2022). Prediction of instructor performance using machine and deep learning techniques. *International Journal of Advanced Computer Science and Applications (IJACSA)* 13, 78–83

Acampora, G. and Cosma, G. (2015). A fuzzy-based approach to programming language independent source-code plagiarism detection. In *2015 IEEE International Conference on Fuzzy Systems (FUZZ-IEEE)* (IEEE), 1–8

Afzal, S., Dhamecha, T. I., Gagnon, P., Nayak, A., Shah, A., Carlstedt-Duke, J., et al. (2020). Ai medical school tutor: Modelling and implementation. In *International Conference on Artificial Intelligence in Medicine* (Springer), 133–145

Agarwal, M. and Mannem, P. (2011). Automatic gap-fill question generation from text books. In *Proceedings of the sixth workshop on innovative use of NLP for building educational applications*. 56–64

Aguiar, E., Chawla, N. V., Brockman, J., Ambrose, G. A., and Goodrich, V. (2014). Engagement vs performance: using electronic portfolios to predict first semester engineering student retention. In *Proceedings of the Fourth International Conference on Learning Analytics And Knowledge*. 103–112

Aguiar, E., Lakkaraju, H., Bhanpuri, N., Miller, D., Yuhas, B., and Addison, K. L. (2015). Who, when, and why: A machine learning approach to prioritizing students at risk of not graduating high school on time. In *Proceedings of the Fifth International Conference on Learning Analytics And Knowledge*. 93–102

Ahmed, A. M., Rizaner, A., and Ulusoy, A. H. (2016). Using data mining to predict instructor performance. *Procedia Computer Science* 102, 137–142

Ahn, J.-w., Tejwani, R., Sundararajan, S., Sipolins, A., O'Hara, S., Paul, A., et al. (2018). Intelligent virtual reality tutoring system supporting open educational resource access. In *International Conference on Intelligent Tutoring Systems* (Springer), 280–286

Alfaro, L., Rivera, C., Luna-Urquizo, J., Castañeda, E., and Fialho, F. (2018). Online learning styles identification model, based on the analysis of user interactions within an e-learning platforms, using neural networks and fuzzy logic. *International Journal of Engineering & Technology* 7, 76

Alfikri, Z. F. and Purwarianti, A. (2014). Detailed analysis of extrinsic plagiarism detection system using machine learning approach (naive bayes and svm). *TELKOMNIKA Indonesian Journal of Electrical Engineering* 12, 7884–7894

AlGhamdi, A., Barsheed, A., AlMshjary, H., and AlGhamdi, H. (2020). A machine learning approach for graduate admission prediction. In *Proceedings of the 2020 2nd International Conference on Image, Video and Signal Processing*. 155–158

Alzahrani, S. (2015). Arabic plagiarism detection using word correlation in n-grams with k-overlapping approach. In *Proceedings of the Workshops at the 7th Forum for Information Retrieval Evaluation (FIRE)*. 123–125

Arroyo, I., Beal, C., Murray, T., Walles, R., and Woolf, B. (2004). Wayang outpost: Intelligent tutoring for high stakes achievement tests. In *Proceedings of the 7th International Conference on Intelligent Tutoring Systems (ITS2004)*. 468–477

Arroyo, I., Cooper, D. G., Burleson, W., and Woolf, B. P. (2010). Bayesian networks and linear regression models of students' goals, moods, and emotions. *Handbook of educational data mining* , 323–338

Arroyo, I., Woolf, B. P., Burelson, W., Muldner, K., Rai, D., and Tai, M. (2014). A multimedia adaptive tutoring system for mathematics that addresses cognition, metacognition and affect. *International Journal of Artificial Intelligence in Education* 24, 387–426

Assiri, B., Bashraheel, M., and Alsuri, A. (2022). Improve the accuracy of students admission at universities using machine learning techniques. In *2022 7th International Conference on Data Science and Machine Learning Applications (CDMA)* (IEEE), 127–132

Bajaj, R. and Sharma, V. (2018). Smart education with artificial intelligence based determination of learning styles. *Procedia computer science* 132, 834–842

Ball, R., Duhadway, L., Feuz, K., Jensen, J., Rague, B., and Weidman, D. (2019). Applying machine learning to improve curriculum design. In *Proceedings of the 50th ACM Technical Symposium on Computer Science Education*. 787–793

Bandara, U. and Wijayarathna, G. (2011). A machine learning based tool for source code plagiarism detection. *International Journal of Machine Learning and Computing* 1, 337

Baral, S., Botelho, A. F., Erickson, J. A., Benachamardi, P., and Heffernan, N. T. (2021). Improving automated scoring of student open responses in mathematics. *International Educational Data Mining Society*

Benedetto, L., Cappelli, A., Turrin, R., and Cremonesi, P. (2020a). Introducing a framework to assess newly created questions with natural language processing. In *International Conference on Artificial Intelligence in Education* (Springer), 43–54

Benedetto, L., Cappelli, A., Turrin, R., and Cremonesi, P. (2020b). R2de: a nlp approach to estimating irt parameters of newly generated questions. In *Proceedings of the Tenth International Conference on Learning Analytics & Knowledge*. 412–421

Bernard, J., Chang, T.-W., Popescu, E., and Graf, S. (2015). Using artificial neural networks to identify learning styles. In *International Conference on Artificial Intelligence in Education* (Springer), 541–544

Botelho, A. F., Baker, R. S., and Heffernan, N. T. (2017). Improving sensor-free affect detection using deep learning. In *International conference on artificial intelligence in education* (Springer), 40–51

Brooks, C., Thompson, C., and Teasley, S. (2015). A time series interaction analysis method for building predictive models of learners using log data. In *Proceedings of the fifth international conference on learning analytics and knowledge*. 126–135

Burstein, J., Chodorow, M., and Leacock, C. (2004). Automated essay evaluation: The criterion online writing service. *Ai magazine* 25, 27–27

Bydžovská, H. (2016). A comparative analysis of techniques for predicting student performance. *International Educational Data Mining Society*

Cen, H., Koedinger, K., and Junker, B. (2006). Learning factors analysis–a general method for cognitive model evaluation and improvement. In *International conference on intelligent tutoring systems* (Springer), 164–175

Chen, Y., Wu, L., and Zaki, M. J. (2020). Reinforcement learning based graph-to-sequence model for natural question generation. In *International Conference on Learning Representations*

Chui, K. T., Liu, R. W., Zhao, M., and De Pablos, P. O. (2020). Predicting students' performance with school and family tutoring using generative adversarial network-based deep support vector machine. *IEEE Access* 8, 86745–86752

Contreras, J. O., Hilles, S., and Abubakar, Z. B. (2018). Automated essay scoring with ontology based on text mining and nltk tools. In *2018 International Conference on Smart Computing and Electronic Enterprise (ICSCEE)* (IEEE), 1–6

Correia, R., Baptista, J., Eskenazi, M., and Mamede, N. (2012). Automatic generation of cloze question stems. In *International Conference on Computational Processing of the Portuguese Language* (Springer), 168–178

Coussement, K., Phan, M., De Caigny, A., Benoit, D. F., and Raes, A. (2020). Predicting student dropout in subscription-based online learning environments: The beneficial impact of the logit leaf model. *Decision Support Systems* 135, 113325

Cummins, R., Zhang, M., and Briscoe, E. (2016). Constrained multi-task learning for automated essay scoring (Association for Computational Linguistics)

Dasgupta, T., Naskar, A., Dey, L., and Saha, R. (2018). Augmenting textual qualitative features in deep convolution recurrent neural network for automatic essay scoring. In *Proceedings of the 5th Workshop on Natural Language Processing Techniques for Educational Applications*. 93–102

Deane, P. and Sheehan, K. (2003). Automatic item generation via frame semantics: Natural language generation of math word problems.

DeBoer, J., Stump, G. S., Seaton, D., Ho, A., Pritchard, D. E., and Breslow, L. (2013). Bringing student backgrounds online: Mooc user demographics, site usage, and online learning. In *Educational data mining 2013*

DeFalco, J. A., Rowe, J. P., Paquette, L., Georgoulas-Sherry, V., Brawner, K., Mott, B. W., et al. (2018). Detecting and addressing frustration in a serious game for military training. *International Journal of Artificial Intelligence in Education* 28, 152–193

Dekker, G. W., Pechenizkiy, M., and Vleeshouwers, J. M. (2009). Predicting students drop out: A case study. *International Working Group on Educational Data Mining*

Dien, T. T., Luu, S. H., Thanh-Hai, N., and Thai-Nghe, N. (2020). Deep learning with data transformation and factor analysis for student performance prediction. *International Journal of Advanced Computer Science and Applications* 11

Doroudi, S. (2019). Integrating human and machine intelligence for enhanced curriculum design. *PhD diss., Air Force Research Laboratory*

Du, X., Shao, J., and Cardie, C. (2017). Learning to ask: Neural question generation for reading comprehension. In *Proceedings of the 55th Annual Meeting of the Association for Computational Linguistics (Volume 1: Long Papers)*. 1342–1352

Duzhin, F. and Gustafsson, A. (2018). Machine learning-based app for self-evaluation of teacher-specific instructional style and tools. *Education Sciences* 8, 7

El Guabassi, I., Bousalem, Z., Marah, R., and Qazdar, A. (2021). A recommender system for predicting students' admission to a graduate program using machine learning algorithms

El Mostafa Hambi, F. B. (2020). A new online plagiarism detection system based on deep learning. *International Journal of Advanced Computer Sciences and Applications* 11, 470–478

El-Rashidy, M. A., Mohamed, R. G., El-Fishawy, N. A., and Shouman, M. A. (2022). Reliable plagiarism detection system based on deep learning approaches. *Neural Computing and Applications* 34, 18837–18858

Esparza, G. G., de Luna, A., Zezzatti, A. O., Hernandez, A., Ponce, J., Álvarez, M., et al. (2017). A sentiment analysis model to analyze students reviews of teacher performance using support vector machines. In *International Symposium on Distributed Computing and Artificial Intelligence* (Springer), 157–164

Fang, J., Zhao, W., and Jia, D. (2019). Exercise difficulty prediction in online education systems. In *2019 International Conference on Data Mining Workshops (ICDMW)* (IEEE), 311–317

Ferrero, J., Besacier, L., Schwab, D., and Agnès, F. (2017). Using word embedding for cross-language plagiarism detection. In *Proceedings of the 15th Conference of the European Chapter of the Association for Computational Linguistics: Volume 2, Short Papers*. 415–421

Ghosh, A., Heffernan, N., and Lan, A. S. (2020). Context-aware attentive knowledge tracing. In *Proceedings of the 26th ACM SIGKDD international conference on knowledge discovery & data mining*. 2330–2339

Gitinabard, N., Xu, Y., Heckman, S., Barnes, T., and Lynch, C. F. (2019). How widely can prediction models be generalized? an analysis of performance prediction in blended courses. *CoRR*

Goh, S. L., Kendall, G., and Sabar, N. R. (2019). Simulated annealing with improved reheating and learning for the post enrolment course timetabling problem. *Journal of the Operational Research Society* 70, 873–888

Goni, M. O. F., Matin, A., Hasan, T., Siddique, M. A. I., Jyoti, O., and Hasnain, F. M. S. (2020). Graduate admission chance prediction using deep neural network. In *2020 IEEE International Women in Engineering (WIE) Conference on Electrical and Computer Engineering (WIECON-ECE)* (IEEE), 259–262

Gordon, A., van Lent, M., Van Velsen, M., Carpenter, P., and Jhala, A. (2004). Branching storylines in virtual reality environments for leadership development. In *Proceedings of the national conference on Artificial Intelligence* (Menlo Park, CA; Cambridge, MA; London; AAAI Press; MIT Press; 1999), 844–851

Gordon, G. and Breazeal, C. (2015). Bayesian active learning-based robot tutor for children's word-reading skills. In *Proceedings of the AAAI Conference on Artificial Intelligence*. vol. 29

Green, D., Walsh, T., Cohen, P., and Chang, Y.-H. (2011). Learning a skill-teaching curriculum with dynamic bayes nets. In *Proceedings of the AAAI Conference on Artificial Intelligence*. vol. 25, 1648–1654

Gupta, D., Vani, K., and Singh, C. K. (2014). Using natural language processing techniques and fuzzy-semantic similarity for automatic external plagiarism detection. In *2014 International Conference on Advances in Computing, Communications and Informatics (ICACCI)* (IEEE), 2694–2699

Gutiérrez, G., Canul-Reich, J., Zezzatti, A. O., Margain, L., and Ponce, J. (2018). Mining: Students comments about teacher performance assessment using machine learning algorithms. *International Journal of Combinatorial Optimization Problems and Informatics* 9, 26

Hänig, C., Remus, R., and De La Puente, X. (2015). Exb themis: Extensive feature extraction from word alignments for semantic textual similarity. In *Proceedings of the 9th international workshop on semantic evaluation (SemEval 2015)*. 264–268

He, J., Bailey, J., Rubinstein, B., and Zhang, R. (2015). Identifying at-risk students in massive open online courses. In *Proceedings of the AAAI Conference on Artificial Intelligence*. vol. 29

Heilman, M. (2011). *Automatic factual question generation from text*. Ph.D. thesis, Carnegie Mellon University

Heilman, M. and Smith, N. A. (2010). Good question! statistical ranking for question generation. In *Human Language Technologies: The 2010 Annual Conference of the North American Chapter of the Association for Computational Linguistics*. 609–617

Herzog, S. (2006). Estimating student retention and degree-completion time: Decision trees and neural networks vis-à-vis regression. *New directions for institutional research* 131, 17–33

Huang, J., Piech, C., Nguyen, A., and Guibas, L. (2013). Syntactic and functional variability of a million code submissions in a machine learning mooc. In *AIED 2013 Workshops Proceedings Volume* (Citeseer), vol. 25

Huang, Y., Huang, W., Tong, S., Huang, Z., Liu, Q., Chen, E., et al. (2021). Stan: Adversarial network for cross-domain question difficulty prediction. In *2021 IEEE International Conference on Data Mining (ICDM)* (IEEE), 220–229

Huang, Y.-T., Tseng, Y.-M., Sun, Y. S., and Chen, M. C. (2014). Tedquiz: automatic quiz generation for ted talks video clips to assess listening comprehension. In *2014 IEEE 14Th international conference on advanced learning technologies* (IEEE), 350–354

Huang, Z., Liu, Q., Chen, E., Zhao, H., Gao, M., Wei, S., et al. (2017). Question difficulty prediction for reading problems in standard tests. In *Thirty-First AAAI Conference on Artificial Intelligence*

Hürlimann, M., Weck, B., van den Berg, E., Suster, S., and Nissim, M. (2015). Glad: Groningen lightweight authorship detection. In *CLEF (Working Notes)*

Idris, N., Yusof, N., Saad, P., et al. (2009). Adaptive course sequencing for personalization of learning path using neural network. *Int. J. Advance. Soft Comput. Appl* 1, 49–61

Imran, A. S., Dalipi, F., and Kastrati, Z. (2019). Predicting student dropout in a mooc: An evaluation of a deep neural network model. In *Proceedings of the 2019 5th International Conference on Computing and Artificial Intelligence*. 190–195

Indurthi, S. R., Raghu, D., Khapra, M. M., and Joshi, S. (2017). Generating natural language question-answer pairs from a knowledge graph using a rnn based question generation model. In *Proceedings of the 15th Conference of the European Chapter of the Association for Computational Linguistics: Volume 1, Long Papers*. 376–385

Islam, M. Z., Ali, R., Haider, A., Islam, M. Z., and Kim, H. S. (2021). Pakes: A reinforcement learning-based personalized adaptability knowledge extraction strategy for adaptive learning systems. *IEEE Access* 9, 155123–155137

Jamison, J. (2017). Applying machine learning to predict davidson college's admissions yield. In *Proceedings of the 2017 ACM SIGCSE Technical Symposium on Computer Science Education*. 765–766

Ji, J.-H., Woo, G., and Cho, H.-G. (2007). A source code linearization technique for detecting plagiarized programs. In *Proceedings of the 12th annual SIGCSE conference on Innovation and technology in computer science education*. 73–77

Jiang, S., Williams, A., Schenke, K., Warschauer, M., and O'dowd, D. (2014). Predicting mooc performance with week 1 behavior. In *Educational data mining 2014*

Jouault, C. and Seta, K. (2013). Building a semantic open learning space with adaptive question generation support. In *Proceedings of the 21st International Conference on Computers in Education*. 41–50

Kalady, S., Elikkottil, A., and Das, R. (2010). Natural language question generation using syntax and keywords. In *Proceedings of QG2010: The Third Workshop on Question Generation* (questiongeneration. org), vol. 2, 5–14

Katta, J. Y. B. (2018). *Machine learning for source-code plagiarism detection*. Ph.D. thesis, International Institute of Information Technology Hyderabad, University of ...

Ke, Z., Inamdar, H., Lin, H., and Ng, V. (2019). Give me more feedback ii: Annotating thesis strength and related attributes in student essays. In *Proceedings of the 57th Annual Meeting of the Association for Computational Linguistics*. 3994–4004

Kenekayoro, P. (2019). Incorporating machine learning to evaluate solutions to the university course timetabling problem. *Covenant Journal of Informatics and Communication Technology*

Khajah, M., Wing, R., Lindsey, R. V., and Mozer, M. (2014). Integrating latent-factor and knowledge-tracing models to predict individual differences in learning. In *EDM*. 99–106

Kim, J. and Shaw, E. (2009). Pedagogical discourse: connecting students to past discussions and peer mentors within an online discussion board. In *Twenty-First IAAI Conference*

Kim, Y., Lee, H., Shin, J., and Jung, K. (2019). Improving neural question generation using answer separation. In *Proceedings of the AAAI conference on artificial intelligence*. vol. 33, 6602–6609

Kloft, M., Stiehler, F., Zheng, Z., and Pinkwart, N. (2014). Predicting mooc dropout over weeks using machine learning methods. In *Proceedings of the EMNLP 2014 workshop on analysis of large scale social interaction in MOOCs*. 60–65

Koncel-Kedziorski, R., Konstas, I., Zettlemoyer, L., and Hajishirzi, H. (2016). A theme-rewriting approach for generating algebra word problems. In *Proceedings of the 2016 Conference on Empirical Methods in Natural Language Processing*. 1617–1628

Lakkaraju, H., Aguiar, E., Shan, C., Miller, D., Bhanpuri, N., Ghani, R., et al. (2015). A machine learning framework to identify students at risk of adverse academic outcomes. In *Proceedings of the 21th ACM SIGKDD international conference on knowledge discovery and data mining*. 1909–1918

Lan, A. S., Vats, D., Waters, A. E., and Baraniuk, R. G. (2015). Mathematical language processing: Automatic grading and feedback for open response mathematical questions. In *Proceedings of the second (2015) ACM conference on learning@ scale*. 167–176

Lange, R. C. and Mancoridis, S. (2007). Using code metric histograms and genetic algorithms to perform author identification for software forensics. In *Proceedings of the 9th annual conference on Genetic and evolutionary computation*. 2082–2089

Li, H., Wang, S., Liang, J., Huang, S., and Xu, B. (2009). High performance automatic mispronunciation detection method based on neural network and trap features. In *Tenth Annual Conference of the International Speech Communication Association*

Li, Q. and Kim, J. (2021). A deep learning-based course recommender system for sustainable development in education. *Applied Sciences* 11, 8993

Li, W., Li, K., Siniscalchi, S. M., Chen, N. F., and Lee, C.-H. (2016). Detecting mispronunciations of l2 learners and providing corrective feedback using knowledge-guided and data-driven decision trees. In *Interspeech*. 3127–3131

Li, X., Chen, M., and Nie, J.-Y. (2020). Sednn: Shared and enhanced deep neural network model for cross-prompt automated essay scoring. *Knowledge-Based Systems* 210, 106491

Lin, C. F., Yeh, Y.-c., Hung, Y. H., and Chang, R. I. (2013). Data mining for providing a personalized learning path in creativity: An application of decision trees. *Computers & Education* 68, 199–210

Lin, L.-H., Chang, T.-H., and Hsu, F.-Y. (2019). Automated prediction of item difficulty in reading comprehension using long short-term memory. In *2019 International Conference on Asian Language Processing (IALP)* (IEEE), 132–135

Lindberg, D., Popowich, F., Nesbit, J., and Winne, P. (2013). Generating natural language questions to support learning on-line. In *Proceedings of the 14th European Workshop on Natural Language Generation*. 105–114

Liu, Q., Huang, Z., Yin, Y., Chen, E., Xiong, H., Su, Y., et al. (2019). Ekt: Exercise-aware knowledge tracing for student performance prediction. *IEEE Transactions on Knowledge and Data Engineering* 33, 100–115

Liu, T., Fang, Q., Ding, W., Li, H., Wu, Z., and Liu, Z. (2021). Mathematical word problem generation from commonsense knowledge graph and equations. In *Proceedings of the 2021 Conference on Empirical Methods in Natural Language Processing*. 4225–4240

Lo, J.-J. and Shu, P.-C. (2005). Identification of learning styles online by observing learners' browsing behaviour through a neural network. *British Journal of Educational Technology* 36, 43–55

Majumder, M. and Saha, S. K. (2015). A system for generating multiple choice questions: With a novel approach for sentence selection. In *Proceedings of the 2nd workshop on natural language processing techniques for educational applications*. 64–72

Manahi, M. S. (2021). *A deep learning framework for the defection of source code plagiarism using Siamese network and embedding models*. Master's thesis, Kuala Lumpur: Kulliyyah of Information and Communication Technology ...

Mardikyan, S. and Badur, B. (2011). Analyzing teaching performance of instructors using data mining techniques. *Informatics in Education* 10, 245–257

Mendis, C., Lahiru, D., Pamudika, N., Madushanka, S., Ranathunga, S., and Dias, G. (2017). Automatic assessment of student answers for geometric theorem proving questions. In *2017 Moratuwa Engineering Research Conference (MERCon)* (IEEE), 413–418

Meuschke, N., Siebeck, N., Schubotz, M., and Gipp, B. (2017). Analyzing semantic concept patterns to detect academic plagiarism. In *Proceedings of the 6th international workshop on mining scientific publications*. 46–53

Mirchi, N., Bissonnette, V., Yilmaz, R., Ledwos, N., Winkler-Schwartz, A., and Del Maestro, R. F. (2020). The virtual operative assistant: An explainable artificial intelligence tool for simulation-based training in surgery and medicine. *PloS one* 15, e0229596

Mokbel, B., Gross, S., Paassen, B., Pinkwart, N., and Hammer, B. (2013). Domain-independent proximity measures in intelligent tutoring systems. In *Educational Data Mining 2013*

Mostafazadeh, N., Misra, I., Devlin, J., Mitchell, M., He, X., and Vanderwende, L. (2016). Generating natural questions about an image. In *Proceedings of the 54th Annual Meeting of the Association for Computational Linguistics (Volume 1: Long Papers)*. 1802–1813

Mota, J. (2008). Using learning styles and neural networks as an approach to elearning content and layout adaptation. In *Doctoral Symposium on Informatics Engineering*

Mothe, J. and Tanguy, L. (2005). Linguistic features to predict query difficulty. In *ACM Conference on research and Development in Information Retrieval, SIGIR, Predicting query difficulty-methods and applications workshop*. 7–10

Movellan, J., Eckhardt, M., Virnes, M., and Rodriguez, A. (2009). Sociable robot improves toddler vocabulary skills. In *Proceedings of the 4th ACM/IEEE international conference on Human robot interaction*. 307–308

Mridha, K., Jha, S., Shah, B., Damodharan, P., Ghosh, A., and Shaw, R. N. (2022). Machine learning algorithms for predicting the graduation admission. In *International Conference on Electrical and Electronics Engineering* (Springer), 618–637

Nadeem, F., Nguyen, H., Liu, Y., and Ostendorf, M. (2019). Automated essay scoring with discourse-aware neural models. In *Proceedings of the fourteenth workshop on innovative use of NLP for building educational applications*. 484–493

Nagatani, K., Zhang, Q., Sato, M., Chen, Y.-Y., Chen, F., and Ohkuma, T. (2019). Augmenting knowledge tracing by considering forgetting behavior. In *The world wide web conference*. 3101–3107

Nakagawa, H., Iwasawa, Y., and Matsuo, Y. (2019). Graph-based knowledge tracing: modeling student proficiency using graph neural network. In *2019 IEEE/WIC/ACM International Conference On Web Intelligence (WI)* (IEEE), 156–163

Nghe, N. T., Janecek, P., and Haddawy, P. (2007). A comparative analysis of techniques for predicting academic performance. In *2007 37th annual frontiers in education conference-global engineering: knowledge without borders, opportunities without passports* (IEEE), T2G–7

Obit, J. H., Landa-Silva, D., Sevaux, M., and Ouelhadj, D. (2011). Non-linear great deluge with reinforcement learning for university course timetabling. *Metaheuristics–Intelligent Decision Making, Series Operations Research/Computer Science Interfaces, Springer* , 1–19

Ohmann, T. and Rahal, I. (2015). Efficient clustering-based source code plagiarism detection using piy. *Knowledge and Information Systems* 43, 445–472

Okoye, K., Arrona-Palacios, A., Camacho-Zuñiga, C., Achem, J. A. G., Escamilla, J., and Hosseini, S. (2022). Towards teaching analytics: a contextual model for analysis of students' evaluation of teaching through text mining and machine learning classification. *Education and Information Technologies* 27, 3891–3933

Olney, A. M., D'Mello, S., Person, N., Cade, W., Hays, P., Williams, C., et al. (2012). Guru: A computer tutor that models expert human tutors. In *International conference on intelligent tutoring systems* (Springer), 256–261

Onan, A. (2020). Mining opinions from instructor evaluation reviews: a deep learning approach. *Computer Applications in Engineering Education* 28, 117–138

Özcan, E., Misir, M., Ochoa, G., and Burke, E. K. (2012). A reinforcement learning: great-deluge hyper-heuristic for examination timetabling. In *Modeling, analysis, and applications in metaheuristic computing: advancements and trends* (IGI Global). 34–55

Pande, C., Witschel, H. F., Martin, A., and Montecchiari, D. (2021). Hybrid conversational ai for intelligent tutoring systems. In *AAAI Spring Symposium: Combining Machine Learning with Knowledge Engineering*

Pandey, S. and Karypis, G. (2019). A self-attentive model for knowledge tracing. In *12th International Conference on Educational Data Mining, EDM 2019* (International Educational Data Mining Society), 384–389

Pavlik Jr, P. I. and Anderson, J. R. (2005). Practice and forgetting effects on vocabulary memory: An activation-based model of the spacing effect. *Cognitive science* 29, 559–586

Pavlik Jr, P. I., Cen, H., and Koedinger, K. R. (2009). Performance factors analysis–a new alternative to knowledge tracing. *Online Submission*

Pereira, J. (2016). Leveraging chatbots to improve self-guided learning through conversational quizzes. In *Proceedings of the fourth international conference on technological ecosystems for enhancing multiculturality*. 911–918

Persing, I., Davis, A., and Ng, V. (2010). Modeling organization in student essays. In *Proceedings of the 2010 conference on empirical methods in natural language processing*. 229–239

Persing, I. and Ng, V. (2013). Modeling thesis clarity in student essays. In *Proceedings of the 51st Annual Meeting of the Association for Computational Linguistics (Volume 1: Long Papers)*. 260–269

Persing, I. and Ng, V. (2014). Modeling prompt adherence in student essays. In *Proceedings of the 52nd Annual Meeting of the Association for Computational Linguistics (Volume 1: Long Papers)*. 1534–1543

Persing, I. and Ng, V. (2015). Modeling argument strength in student essays. In *Proceedings of the 53rd Annual Meeting of the Association for Computational Linguistics and the 7th International Joint Conference on Natural Language Processing (Volume 1: Long Papers)*. 543–552

Pertile, S. d. L., Moreira, V. P., and Rosso, P. (2016). Comparing and combining c ontent-and c itation-based approaches for plagiarism detection. *Journal of the Association for Information Science and Technology* 67, 2511–2526

Phandi, P., Chai, K. M. A., and Ng, H. T. (2015). Flexible domain adaptation for automated essay scoring using correlated linear regression. In *Proceedings of the 2015 Conference on Empirical Methods in Natural Language Processing*. 431–439

Piech, C., Bassen, J., Huang, J., Ganguli, S., Sahami, M., Guibas, L. J., et al. (2015a). Deep knowledge tracing. *Advances in neural information processing systems* 28

Piech, C., Huang, J., Nguyen, A., Phulsuksombati, M., Sahami, M., and Guibas, L. (2015b). Learning program embeddings to propagate feedback on student code. In *International conference on machine Learning* (PMLR), 1093–1102

Polozov, O., O'Rourke, E., Smith, A. M., Zettlemoyer, L., Gulwani, S., and Popović, Z. (2015). Personalized mathematical word problem generation. In *Twenty-Fourth International Joint Conference on Artificial Intelligence*

Pu, Y., Wang, C., and Wu, W. (2020). A deep reinforcement learning framework for instructional sequencing. In *2020 IEEE International Conference on Big Data (Big Data)* (IEEE), 5201–5208

Qiu, Z., Wu, X., and Fan, W. (2019). Question difficulty prediction for multiple choice problems in medical exams. In *Proceedings of the 28th ACM International Conference on Information and Knowledge Management*. 139–148

Ramesh, A., Goldwasser, D., Huang, B., Daumé III, H., and Getoor, L. (2013). Modeling learner engagement in moocs using probabilistic soft logic. In *NIPS workshop on data driven education*. vol. 21, 62

Ramesh, A., Goldwasser, D., Huang, B., Daume III, H., and Getoor, L. (2014). Learning latent engagement patterns of students in online courses. In *Twenty-eighth AAAI conference on artificial intelligence*

Rawatlal, R. (2017). Application of machine learning to curriculum design analysis. In *2017 Computing Conference* (IEEE), 1143–1151

Reddy, S., Levine, S., and Dragan, A. (2017). Accelerating human learning with deep reinforcement learning. In *NIPS'17 Workshop: Teaching Machines, Robots, and Humans*. 5–9

Shashidhar, V., Pandey, N., and Aggarwal, V. (2015). Automatic spontaneous speech grading: A novel feature derivation technique using the crowd. In *Proceedings of the 53rd Annual Meeting of the Association for Computational Linguistics and the 7th International Joint Conference on Natural Language Processing (Volume 1: Long Papers)*. 1085–1094

Shen, S., Liu, Q., Chen, E., Huang, Z., Huang, W., Yin, Y., et al. (2021). Learning process-consistent knowledge tracing. In *Proceedings of the 27th ACM SIGKDD Conference on Knowledge Discovery & Data Mining*. 1452–1460

Singh, G., Srikant, S., and Aggarwal, V. (2016). Question independent grading using machine learning: The case of computer program grading. In *Proceedings of the 22nd ACM SIGKDD International Conference on Knowledge Discovery and Data Mining*. 263–272

Sinha, T., Li, N., Jermann, P., and Dillenbourg, P. (2014). Capturing "attrition intensifying" structural traits from didactic interaction sequences of mooc learners. *EMNLP 2014* , 42

Soleman, S. and Purwarianti, A. (2014). Experiments on the indonesian plagiarism detection using latent semantic analysis. In *2014 2nd International Conference on Information and Communication Technology (ICoICT)* (IEEE), 413–418

Somasundaram, M., Latha, P., and Pandian, S. S. (2020). Curriculum design using artificial intelligence (ai) back propagation method. *Procedia Computer Science* 172, 134–138

Song, W., Zhang, K., Fu, R., Liu, L., Liu, T., and Cheng, M. (2020). Multi-stage pre-training for automated chinese essay scoring. In *Proceedings of the 2020 Conference on Empirical Methods in Natural Language Processing (EMNLP)*. 6723–6733

Srikant, S. and Aggarwal, V. (2014). A system to grade computer programming skills using machine learning. In *Proceedings of the 20th ACM SIGKDD international conference on Knowledge discovery and data mining*. 1887–1896

Stiller, J., Hartmann, S., Mathesius, S., Straube, P., Tiemann, R., Nordmeier, V., et al. (2016). Assessing scientific reasoning: A comprehensive evaluation of item features that affect item difficulty. *Assessment & Evaluation in Higher Education* 41, 721–732

Su, Y., Liu, Q., Liu, Q., Huang, Z., Yin, Y., Chen, E., et al. (2018). Exercise-enhanced sequential modeling for student performance prediction. In *Proceedings of the AAAI Conference on Artificial Intelligence*. vol. 32

Sultan, M. A., Bethard, S., and Sumner, T. (2014). Dls @ cu: Sentence similarity from word alignment. In *SemEval@ COLING*. 241–246

Taghipour, K. and Ng, H. T. (2016). A neural approach to automated essay scoring. In *Proceedings of the 2016 conference on empirical methods in natural language processing*. 1882–1891

Tamhane, A., Ikbal, S., Sengupta, B., Duggirala, M., and Appleton, J. (2014). Predicting student risks through longitudinal analysis. In *Proceedings of the 20th ACM SIGKDD international conference on Knowledge discovery and data mining*. 1544–1552

Tan, S., Doshi-Velez, F., Quiroz, J., and Glassman, E. (2017). Clustering latex solutions to machine learning assignments for rapid assessment

Taoum, J., Nakhal, B., Bevacqua, E., and Querrec, R. (2016). A design proposition for interactive virtual tutors in an informed environment. In *International Conference on Intelligent Virtual Agents* (Springer), 341–350

Tarcsay, B., Vasić, J., and Perez-Tellez, F. (2022). Use of machine learning methods in the assessment of programming assignments. In *International Conference on Text, Speech, and Dialogue* (Springer), 151–159

Thai-Nghe, N., Drumond, L., Krohn-Grimberghe, A., and Schmidt-Thieme, L. (2010). Recommender system for predicting student performance. *Procedia Computer Science* 1, 2811–2819

Tong, S., Liu, Q., Huang, W., Hunag, Z., Chen, E., Liu, C., et al. (2020). Structure-based knowledge tracing: an influence propagation view. In *2020 IEEE International Conference on Data Mining (ICDM)* (IEEE), 541–550

Toscher, A. and Jahrer, M. (2010). Collaborative filtering applied to educational data mining. *KDD cup*

Trakunphutthirak, R., Cheung, Y., and Lee, V. C. (2019). A study of educational data mining: Evidence from a thai university. In *Proceedings of the AAAI Conference on Artificial Intelligence*. vol. 33, 734–741

Tschuggnall, M. and Specht, G. (2013). Detecting plagiarism in text documents through grammar-analysis of authors. In *BTW*. 241–259

Tsiakmaki, M., Kostopoulos, G., Kotsiantis, S., and Ragos, O. (2020). Transfer learning from deep neural networks for predicting student performance. *Applied Sciences* 10, 2145

Ullah, F., Jabbar, S., and Mostarda, L. (2021). An intelligent decision support system for software plagiarism detection in academia. *International Journal of Intelligent Systems* 36, 2730–2752

Upadhyay, U., De, A., and Gomez Rodriguez, M. (2018). Deep reinforcement learning of marked temporal point processes. *Advances in Neural Information Processing Systems* 31

Uto, M. and Okano, M. (2020). Robust neural automated essay scoring using item response theory. In *International Conference on Artificial Intelligence in Education* (Springer), 549–561

Vani, K. and Gupta, D. (2014). Using k-means cluster based techniques in external plagiarism detection. In *2014 international conference on contemporary computing and informatics (IC3I)* (IEEE), 1268–1273

Vijayalakshmi, V., Panimalar, K., and Janarthanan, S. (2020). Predicting the performance of instructors using machine learning algorithms. *High Technology Letters* 26

Villaverde, J. E., Godoy, D., and Amandi, A. (2006). Learning styles' recognition in e-learning environments with feed-forward neural networks. *Journal of Computer Assisted Learning* 22, 197–206

Vujošević-Janičić, M., Nikolić, M., Tošić, D., and Kuncak, V. (2013). Software verification and graph similarity for automated evaluation of students' assignments. *Information and Software Technology* 55, 1004–1016

Waheed, H., Hassan, S.-U., Aljohani, N. R., Hardman, J., Alelyani, S., and Nawaz, R. (2020). Predicting academic performance of students from vle big data using deep learning models. *Computers in Human behavior* 104, 106189

Wang, K. and Su, Z. (2015). Automated geometry theorem proving for human-readable proofs. In *Twenty-Fourth International Joint Conference on Artificial Intelligence*

Wang, L., Sy, A., Liu, L., and Piech, C. (2017). Learning to represent student knowledge on programming exercises using deep learning. *International Educational Data Mining Society*

Wang, T., Su, X., Wang, Y., and Ma, P. (2007). Semantic similarity-based grading of student programs. *Information and Software Technology* 49, 99–107

Wang, Z., Lan, A., and Baraniuk, R. (2021). Math word problem generation with mathematical consistency and problem context constraints. In *2021 Conference on Empirical Methods in Natural Language Processing*

Waters, A. and Miikkulainen, R. (2014). Grade: Machine learning support for graduate admissions. *Ai Magazine* 35, 64–64

Wen, M., Yang, D., and Rose, C. (2014). Sentiment analysis in mooc discussion forums: What does it tell us? In *Educational data mining 2014*

Woolf, B. P., Arroyo, I., Muldner, K., Burleson, W., Cooper, D. G., Dolan, R., et al. (2010). The effect of motivational learning companions on low achieving students and students with disabilities. In *International conference on intelligent tutoring systems* (Springer), 327–337

Wu, M., Mosse, M., Goodman, N., and Piech, C. (2019). Zero shot learning for code education: Rubric sampling with deep learning inference. In *Proceedings of the AAAI Conference on Artificial Intelligence*. vol. 33, 782–790

Wu, Q., Zhang, Q., and Huang, X. (2022). Automatic math word problem generation with topic-expression co-attention mechanism and reinforcement learning. *IEEE/ACM Transactions on Audio, Speech, and Language Processing* 30, 1061–1072

Xu, J., Han, Y., Marcu, D., and Van Der Schaar, M. (2017). Progressive prediction of student performance in college programs. In *Thirty-First AAAI Conference on Artificial Intelligence*

Xue, K., Yaneva, V., Runyon, C., and Baldwin, P. (2020). Predicting the difficulty and response time of multiple choice questions using transfer learning. In *Proceedings of the Fifteenth Workshop on Innovative Use of NLP for Building Educational Applications*. 193–197

Yaneva, V., Baldwin, P., Mee, J., et al. (2020). Predicting item survival for multiple choice questions in a high-stakes medical exam. In *Proceedings of The 12th Language Resources and Evaluation Conference*. 6812–6818

Yang, D., Sinha, T., Adamson, D., and Rosé, C. P. (2013). Turn on, tune in, drop out: Anticipating student dropouts in massive open online courses. In *Proceedings of the 2013 NIPS Data-driven education workshop*. vol. 11, 14

Yang, Y., Shen, J., Qu, Y., Liu, Y., Wang, K., Zhu, Y., et al. (2020). Gikt: a graph-based interaction model for knowledge tracing. In *Joint European Conference on Machine Learning and Knowledge Discovery in Databases* (Springer), 299–315

Young, N. and Caballero, M. (2019). Using machine learning to understand physics graduate school admissions. In *Proceedings of the Physics Education Research Conference (PERC*. 669–674

Yudelson, M. V., Koedinger, K. R., and Gordon, G. J. (2013). Individualized bayesian knowledge tracing models. In *International conference on artificial intelligence in education* (Springer), 171–180

Zatarain-Cabada, R., Barrón-Estrada, M. L., Angulo, V. P., García, A. J., and García, C. A. R. (2010). A learning social network with recognition of learning styles using neural networks. In *Mexican Conference on Pattern Recognition* (Springer), 199–209

Zhang, H., Magooda, A., Litman, D., Correnti, R., Wang, E., Matsmura, L., et al. (2019). erevise: Using natural language processing to provide formative feedback on text evidence usage in student writing. In *Proceedings of the AAAI conference on artificial intelligence*. vol. 33, 9619–9625

Zhang, J., Shi, X., King, I., and Yeung, D.-Y. (2017). Dynamic key-value memory networks for knowledge tracing. In *Proceedings of the 26th international conference on World Wide Web*. 765–774

Zhang, L., Zhao, Z., Ma, C., Shan, L., Sun, H., Jiang, L., et al. (2020). End-to-end automatic pronunciation error detection based on improved hybrid ctc/attention architecture. *Sensors* 20, 1809

Zhang, M., Baral, S., Heffernan, N., and Lan, A. (2022). Automatic short math answer grading via in-context meta-learning. In *Proceedings of the International Conference on Educational Data Mining*

Zhao, Y., Lackaye, B., Dy, J. G., and Brodley, C. E. (2020). A quantitative machine learning approach to master students admission for professional institutions. *International Educational Data Mining Society*

Zhao, Y., Ni, X., Ding, Y., and Ke, Q. (2018). Paragraph-level neural question generation with maxout pointer and gated self-attention networks. In *Proceedings of the 2018 conference on empirical methods in natural language processing*. 3901–3910

Zhou, Q. and Huang, D. (2019). Towards generating math word problems from equations and topics. In *Proceedings of the 12th International Conference on Natural Language Generation*. 494–503
